# Prevention of white spot lesions using three remineralizing agents: An in vitro comparative study

**DOI:** 10.15171/joddd.2019.006

**Published:** 2019-04-24

**Authors:** Soodeh Tahmasbi, Seyedezahra Mousavi, Marjan Behroozibakhsh, Mohammadreza Badiee

**Affiliations:** ^1^Department of Orthodontics, Preventive Dentistry Research Center, Research Institute of Dental Sciences, Dental School, Shahid Beheshti University of Medical Sciences, Tehran, Iran; ^2^Dentist, 3Dentofacial Deformities Research Center, Research Institute of Dental Sciences, Shahid Beheshti University of Medical Sciences, Tehran, Iran; ^3^Department of Dental Materials, School of Dentistry, Tehran University of Medical Sciences, Tehran, Iran; ^4^Dentofacial Deformities Research Center, Research Institute of Dental Sciences, Shahid Beheshti University of Medical Sciences, Tehran, Iran

**Keywords:** Dental caries, fluorides, orthodontics, tooth demineralization

## Abstract

***Background***. Enamel demineralization around orthodontic brackets is an important clinical problem. This study sought to compare the efficacy of sodium fluoride (NaF), casein phosphopeptide amorphous calcium phosphate fluoride (CPP-ACP-F; MI Paste Plus) and a water-based cream (Remin Pro), which contains hydroxyapatite and fluoride for prevention of enamel demineralization.

***Methods***. Fifty-six sound human premolars extracted for orthodontic purposes were collected. After cleaning, the crowns were mounted in acrylic resin and all the surfaces were coated with nail varnish except for a 3×4-mm window on the buccal surface. The samples were randomly divided into four groups of 14 and subjected to pH cycling for 14 days, during which the teeth were immersed in artificial saliva for 21 hours and in demineralizing agent for three hours daily. Before transferring the samples from the saliva to the demineralizing solution, the remineralizing agent (0.05% NaF, MI Paste Plus or Remin Pro Paste, depending on the group) was applied on the samples once a day for five minutes. No remineralizing agent was used in the control group. Surface microhardness of samples was measured by Vickers microhardness tester at baseline and after the intervention. The data were analyzed using one-way ANOVA, ANCOVA, Bonferroni test and Tukey test. Statistical significance was set at P<0.05.

***Results***. The mean microhardness was significantly different between the test and control groups (P<0.0001). Other differences were not significantly different (P>0.05).

***Conclusion***. The results showed that 0.05% NaF was more efficient than Remin Pro and MI Paste Plus for prevention of white spot lesions (WSLs). Remin Pro and MI Paste Plus were not significantly difference from the control group in this regard.

## Introduction

Enamel demineralization around orthodontic brackets is an important clinical problem. The prevalence of white spot lesions (WSLs) in orthodontic patients ranges from 25% to 46%.^1-3^ These lesions most commonly occur in the cervical part of the middle third of the crowns of first molar, lateral incisor and canine teeth.^4^ White spot lesions can be detected as white opaque lesions after air-drying the teeth.^5^ These lesions often develop after four weeks if no anticariogenic agent is used, which highlights their fast occurrence.^6^ High prevalence of enamel decalcification during fixed orthodontic treatment is partly attributed to the irregular bracket surface and presence of‏ orthodontic wires, bands and other attachments, which enhance plaque retention, complicate oral hygiene and limit the self-cleaning capacity of teeth with the salivary flow and movement of oral muscles. Consequently, the plaque pH drops due to the presence of fermentable carbohydrates, faster accumulation and maturation of plaque and colonization of aciduric bacteria such as *Streptococcus mutans* and *Lactobacilli*.^7^ Elimination of microbial plaque after orthodontic treatment is not sufficient for treatment of WSLs because secondary lesions may develop 5‒12 years after completion of orthodontic treatment.^8^ On the other hand, natural remineralization by the mineral ions present in the saliva only occurs in the superficial layer of WSLs. Moreover, even after remineralization, the appearance of the tooth is not esthetically acceptable. Thus, remineralizing agents are required for treatment of deeper lesions and improved esthetics.^7^ Control of WSLs includes prevention of demineralization and enhancement of remineralization of primary lesions. Obviously, prevention is superior to treatment.^7^ No consensus has been reached on an efficient, predictable and esthetically acceptable treatment for these lesions.^4^ The most important preventive measure is to increase enamel resistance against acid attacks.^9^ The ability of bacterial biofilm to absorb calcium, phosphate and fluoride from the saliva and extraoral sources results in enamel remineralization following demineralization. Efficient remineralization requires the exposure of enamel to low concentrations of the above-mentioned ions for long periods of time. Thus, extraoral sources of calcium, phosphate and fluoride can change the cariogenic potential of dental biofilm.^7^ Fluoride is the most commonly used substance for remineralization.^5^ The scientific basis for the use of fluoride in fight against caries is that fluoride ions can penetrate into the crystalline structure of dental hard tissues, decrease their solubility and confer acid resistance. Fluoride ions replace the hydroxyl groups in the formulation of hydroxyapatite and result in the formation of fluorapatite.^1^ Fluoride inhibits further demineralization of enamel but at the same time, it prevents the uptake of calcium and phosphate ions, which are required for the repair of deep lesions.^5^

MI Paste Plus contains amorphous calcium phosphate (ACP), casein phosphopeptide (CPP) and fluoride; ACP is a reactive, super-saturated solution of calcium and phosphate, which can release these ions as well as α s1-casein and β-casein. The ACP-CPP nanocomplex can penetrate into the enamel porosities due to the small size of particles. It remineralizes the superficial enamel crystals and prevents demineralization of tooth structure.^10^

Remin Pro, a new remineralizing agent, was recently introduced to the market. It is supplied in the form of a water-based cream, which contains hydroxyapatite and fluoride. It has been suggested for prevention of enamel demineralization during the course of orthodontic treatment and after dental bleaching to prevent and control tooth hypersensitivity. It has been claimed that this product is preferred for use by patients who are allergic to bovine proteins since it is devoid of bovine proteins present in the composition of CPP-ACP. In search of the literature, no previous study was found to have compared the efficacy of Remin Pro and MI Paste Plus or fluoride for prevention of WSLs.^11^ Thus, this in vitro study was conducted to compare the efficacy of NaF, MI Paste Plus and Remin Pro for prevention of enamel demineralization.

## Methods

Ethical approval (code: IR.SBMU.RIDS.REC.1394.60) was obtained from Shahid Beheshti University Medical Ethics Committee. Fifty-six sound human premolars (without discoloration or coronal caries) were collected and cleaned with pumice paste and prophy brush using a low-speed handpiece. The crowns were cut at the cementoenamel junction using a fissure bur and a high-speed handpiece. All the samples were stored in saline solution. Using a sticker, a window measuring 3×4 mm was covered on the buccal surface of the samples and the remaining areas were coated with nail varnish. The samples were then mounted in auto-polymerizing acrylic resin (Acropars, Tehran, Iran) and coded from 1 to 56. Prior to the intervention, the microhardness of samples was measured using a microhardness tester (V-Test, Baresiss, Germany) in three points with a Vickers diamond indenter under a load of 200 N for 10 seconds. The resultant value was recorded for each sample. Next, each sample was immersed in saline solution in a glass container. The samples were randomly divided into four groups of 14 and subjected to the intervention for 14 days. The four groups included the control group (group 1), NaF (group 2), MI Paste Plus (group 3) and Remin Pro (group 4).

During the experiment, two solutions (manufactured in the chemical laboratory of Jahad Daneshgahi, Tehran, Iran) were used for testing the samples:

1. A demineralizing solution with a pH of 4.5 and the following formulation: CaCl2 (2.2 mM) + NaH_2_PO_4_ (2.2 mM) + acetic acid (50 mM) + NaCl (100 mM) + NaF (1 ppm) + NaN_3_ (0.02%).
2. Artificial saliva with a pH of 6.9 with the following formulation: KCl (0.04) + NaCl (0.04) + CaCl_2_.2H_2_ (0.09) + NaH_2_PO_4_.2H_2_ (0.069) + MgCl_2_.6H_2_O (0.008) + urea (0.05) + ascorbic acid (0.01) + H_2_O (10 mL + methyl-p hydroxyl benzoate (0.15) +  PEG 6000 (7) + glucose (0.1)

First, the stock solution was prepared from the above-mentioned compounds. Next, using the formula C1V1=C2V2, the required volume of each solution was determined. Afterwards, the samples were subjected to pH cycling for 14 days as follows:

1. Control group: The samples in this group (#1 to #14) were immersed in 10 mL of artificial saliva for 21 hours daily and then immersed in 10 mL of demineralizing agent for three hours. It should be noted that these solutions were refreshed daily.
2. 0.05% NaF group: The samples in this group (#15 to #28) were immersed in 10 mL of artificial saliva for 21 hours daily and then immersed in 0.05% NaF (manufactured in the chemical laboratory of Jahad Daneshgahi, Tehran, Iran) for five minutes. Next, the samples were retrieved from the mouthwash and immersed in the demineralizing agent for three hours.
3. MI Paste Plus group: The samples in this group (#29 to #42) were immersed in 10 mL of artificial saliva for 21 hours daily. Using a microbrush, MI Paste Plus (Recaldent, GC Corp., Japan) was then applied on their surfaces. After five minutes (as recommended by the manufacturer), the paste was wiped off the surfaces (without rinsing with water) and the samples were placed in the demineralizing solution for three hours.
4. Remin Pro group: The samples in this group (#43 to #56) were treated similar to that in the MI Paste Plus group with the exception that Remin Pro (Voco, Germany) was used as the remineralizing agent instead of MI Paste Plus. It should be noted that the samples were not washed during the process of transfer from one solution to the other.

After 14 days, microhardness of samples was measured using a microhardness tester (V-Test, Baresiss, Germany) in three points with a Vickers diamond indenter under a load of 200 N for 10 seconds under the same conditions. The microhardness values were recorded for each sample.

### Statistical Analysis

To assess the presence of significant differences among the groups, data were analyzed using one-way ANOVA and Tukey test. Statistical analysis of the data was carried out using SPSS 21. Statistical significance was set at P<0.05.

## Results

**Figure 1:**
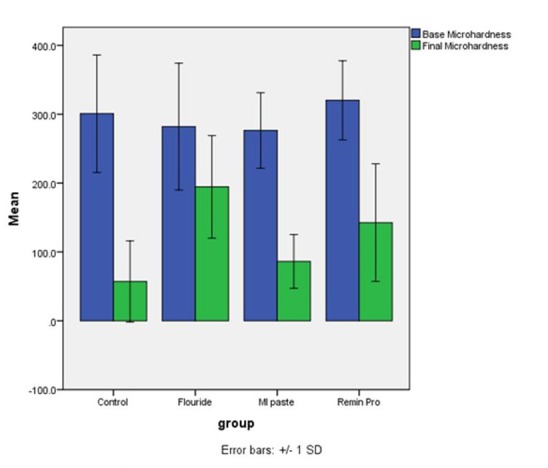
Comparison of changes in surface microhardness after the intervention compared to baseline in the four groups.

Assessment of microhardness values after the intervention revealed that the surface microhardness of all the samples in the four groups changed after the intervention compared to the baseline values. The minimum change in microhardness occurred in group 2 samples subjected to NaF mouthwash, which means that NaF had the greatest efficacy for prevention of enamel demineralization, followed by Remin Pro (group 4), MI Paste Plus (group 3) and the control group (group 1). Descriptive statistics of surface microhardness values in the study groups are shown in Table 1. The mean changes in microhardness of samples are presented in Figure 1.

**Table 1:** Descriptive statistics (Mean (SD)) of surface microhardness in the four groups

**Study groups**	**Baseline microhardness**	**Post-intervention microhardness**	**Change in microhardness**	**Relative change**
**Control**	300.82 (85.25)	57.08 (58.93)	243.73 (85.47)	0.81 (0.15)
**0.05% NaF**	282.14 (92.05)	194.54 (74.41)	87.6 (91.55)	0.24 (0.31)
**MI Paste Plus**	276.50 (54.88)	86.25 (39.05)	190.25 (69.04)	0.67 (0.14)
**Remin Pro**	314.0 (59.41)	148.05 (84.19)	165.95 (81.14)	0.53 (0.26)

**Figure 2:**
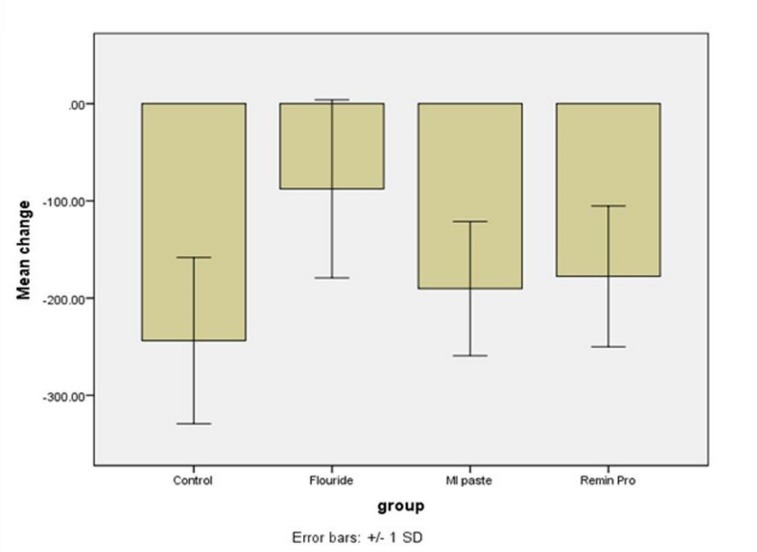
Relative changes in surface microhardness.

Changes in surface microhardness of the samples after intervention was compared among the four groups using one-way ANOVA, which showed significant differences in microhardness changes between two or more groups (P<0.05, Figure 2). Thus, pairwise comparisons of the groups were carried out using Tukey tests. The differences in the mean changes in surface microhardness were significant between the NaF and control (P<0.0001), NaF and MI Paste Plus (P=0.01) and NaF and Remin Pro (P=0.03) groups; the remaining differences between the groups were not statistically significant (P>0.05).

## Discussion

White spot lesions are subsurface enamel porosities, which develop due to enamel demineralization. WSLs often occur at the center of tooth surface and have a distinguishable margin. The WSLs are highly prevalent in patients under fixed orthodontic treatment due to the presence of orthodontic wires and bands, which enhance plaque retention, decrease pH and result in rapid shift of oral microbial flora to a pathogenic flora.^12^

Although several methods have been proposed for prevention and control of initial enamel lesions, no consensus has been reached on an ideal solution for this common problem. As a result, WSLs remain a major concern for orthodontists and patients. Considering the high prevalence of WSLs in orthodontic patients and the significance of esthetics in these patients, occurrence of WSLs must be prevented. Thus, we evaluated the efficacy of NaF, MI Paste Plus and Remin Pro for prevention of WSLs to find the most efficient agent for this purpose. The second objective of this study was to compare the efficacy of Remin Pro, a recently introduced agent, with the remaining two agents. It contains hydroxyapatite and fluoride and can decrease tooth hypersensitivity, prevent enamel demineralization and enhance remineralization of enamel lesions.^13^

In a systematic review by Chen et al,^8^ the efficacy of remineralizing agents for the treatment of WSLs after orthodontic treatment, CPP-ACP, NaF and MI Paste Plus were introduced as effective agents for treatment of enamel lesions. However, comparison of the results of the reviewed studies yielded no definite conclusion regarding the most ideal and reliable agent for treatment of primary enamel lesions.

In this study, pH cycling was performed to simulate the oral environment and assess the preventive effect of agents. Duration of immersion of samples in the demineralizing agent and artificial saliva has been variable in different studies between 5 to 28 consecutive days, 3 to 6 hours a day in demineralizing agent.^14-16^ In this study, we immersed the samples for 21 hours a day in artificial saliva with a pH of 6.9 and in demineralizing agent with a pH of 4.5 for three hours for a period of 14 days. The pH of demineralizing agent in previous studies varied from 3.5 to 5.^17,18^

Several methods have been used to assess the degree of remineralization of samples, including DIAGNOdent,^13^ quantitative light-induced fluorescence (QLF), scanning electron microscopy,^19^ polarized light microscope,^20-21^ x-ray spectrophotometer,^9^ standard photography,^4^ micro-computed tomography^22^ and transverse micro-radiography.^23^

Microhardness tester has been used in a number of studies for assessment of surface microhardness of samples.^5,15,16,24^ Similarly, we used Vickers microhardness tester for measurement of surface microhardness of samples in three points; 200-N load was applied for 10 seconds at baseline and after the intervention for this purpose.

It should be mentioned that many previous studies on prevention of WSLs have evaluated the efficacy of different orthodontic bracket bonding agents for prevention of enamel lesions.^24-26^ A limited number of studies have compared the caries prevention efficacy of CPP-ACP and MI Paste Plus with a methodology similar to ours; however, the therapeutic and remineralizing effects of these products have been well documented.^4,5,8^

Assessment of the microhardness values obtained in the current study revealed that minimal changes occurred in microhardness of the NaF group, which indicates that among the study groups, NaF had the greatest efficacy for prevention of enamel demineralization. Remin Pro, MI Paste Plus and control group ranked next. This finding was in agreement with the results of several previous studies. Hamba et al^22^ reported minimum loss of minerals and shallowest lesions in the fluoride group compared to CPP-ACP and CPP-ACP-F.^22^ The preventive effects of CPP-ACP, MI Paste Plus, fluoride varnish and sodium-calcium-phosphosilicate were compared in another study and it was found that fluoride varnish was the most effective of all.^21^ Another study confirmed the optimal efficacy of fluoride for prevention of WSLs.^27^ Several clinical and in vitro studies have documented that fluoride-containing products have the greatest remineralizing effect compared to other products.^15^

Bergstrand and Twetman^28^ collected evidence on the efficacy of primary and secondary (therapeutic) preventive measures for WSLs around orthodontic brackets. In seven out of nine articles related to prevention, the mean preventive effect of fluoride was reported to be 42.5%. An in vitro study assessed the efficacy of four types of bioactive glass bonding agents compared to Transbond XT conventional bonding agent for prevention of WSLs and concluded that fluoride-containing bonding agents was most effective for prevention of WSLs.^14^ Another study compared the efficacy of five different agents for prevention of WSLs and concluded that fluoride-containing glass-ionomers significantly prevented the occurrence of sub-surface lesions around orthodontic brackets.^19^

In the current study, Remin Pro ranked second after NaF and prevented the occurrence of enamel lesions to some extent but had no significant difference from the control group. A search in the literature yielded only one study on the remineralizing effect of Remin Pro, conducted in 2014, which assessed the changes in mineral content of enamel surface due to the application of acidulated phosphate fluoride, Remin Pro and propolis along with CO_2_ laser irradiation showed the highest mineral content in samples treated with acidulated phosphate fluoride combined with laser irradiation.^9^ This finding was in agreement with our results, indicating that fluoride products were more effective than Remin Pro in caries prevention.

The majority of previous studies on Remin Pro focused on its effects on bleached teeth. For instance, Heshmat et al^29^ evaluated and compared the effect of Remin Pro, MI Paste Plus and natural saliva on microhardness of bleached teeth. They measured the microhardness at baseline and after bleaching with 35% hydrogen peroxide. Next, they used Remin Pro, MI Paste Plus and natural saliva for 15 days and measured the microhardness of samples again after the intervention. They concluded that the surface microhardness of samples decreased after bleaching but no significant difference was noted among the three materials for increasing the surface microhardness of samples.

Despite the numerous advantages of CPP-ACP and MI Paste Plus, results of studies on their efficacy for prevention of enamel demineralization are controversial. However, the therapeutic effects of these agents have been previously confirmed and it has been stated that materials such as CPP-ACP paste have therapeutic efficacy almost similar to that of NaF.^5,16,24,27^ In the current study, MI Paste Plus ranked third after NaF and Remin Pro. MI Paste Plus had insignificant efficacy for prevention of enamel demineralization and had no significant difference from the control group in this respect. However, in some previous studies, MI Paste Plus significantly inhibited the formation of enamel lesions, although its efficacy was still lower than that of fluoride in this respect.^21-23^ This controversy in the results might be attributed to differences in methods of assessment of samples since we only measured the surface microhardness using a Vickers microhardness tester while previous studies used polarized light microscope and micro-computed tomography for precise quantification of mineral loss and measurement of depth of lesions.^21,23^

## Conclusion

This in vitro study showed that NaF mouthwash had the greatest efficacy for prevention of enamel demineralization compared with Remin Pro and MI Paste Plus. Although the latter two inhibited enamel demineralization to some extent, they exhibited no significant difference from the control group in this regard.

## Conflict of Interests

The authors declare no conflict(s) of interest related to the publication of this work.

## Author contributions

1- Design of the work: Soodeh Tahmasbi
2- The acquisition: Seyedezahra Mousavi, Marjan Behroozibakhsh
3- Analysis: Mohammadreza Badiee, Soodeh Tahmasbi
4- Drafting the work: Mohammadreza Badiee

## Acknowledgements

The authors would like to thank the Research Deputy of the Dental School at Shahid Beheshti University of Medical Sciences for their grant and financial support. This study was extracted from the undergraduate thesis of Seyedezahra Mousavi under supervision of Dr. Soodeh Tahmasbi at Shahid Beheshti Dental School.

## Funding

This study was funded by the Research Deputy of the Dental School, Shahid Beheshti University of Medical Sciences.

## Ethics approval

This study was approved by ethics committee of Research Institute of Dental scinces, Shahid Beheshti University of Medical Sciences and the reference number is IR.SBMU.RIDS.REC.1394.69.
